# Economic Considerations in COVID‐19 Vaccine Hesitancy and Refusal: A Survey of the Literature*

**DOI:** 10.1111/1475-4932.12667

**Published:** 2022-05-24

**Authors:** Louise Rawlings, Jeffrey C. L. Looi, Stephen J. Robson

**Affiliations:** ^1^ Crawford School of Public Policy Canberra Australian Capital Territory Australia; ^2^ College of Health and Medicine Australian National University Garran Australian Capital Territory Australia

## Abstract

The COVID‐19 global pandemic has triggered one of the greatest economic shocks in a century. Effective COVID‐19 vaccines have been developed, but a proportion of people either are hesitant or refuse to be vaccinated, facilitated by a global misinformation campaign. If ‘herd immunity’ cannot be achieved, there is potential not only for ongoing surges in infection, but also for development of new strains of the virus that could evade vaccines and precipitate further health and economic crises. We review the economics of vaccination and of vaccine hesitancy and refusal, and their potential effects on the recovery from the COVID‐19 pandemic.

## I Introduction

Coronavirus disease 2019 (COVID‐19) was first described in the city of Wuhan, China, in December of 2019 and subsequently spread rapidly across the world: at the time of writing (September, 2021) only five small island nations had not reported infections.^1^ COVID‐19 results from infection with the severe acute respiratory syndrome coronavirus 2 (SARS‐CoV‐2), a new type of coronavirus. Coronaviruses – so‐named because their surface spike proteins given them the appearance of a crown – are the cause of the common cold and are known to be highly infectious. At the time of writing the World Health Organisation (WHO) reported a total of almost 225 million confirmed cases globally, with over 4.6 million deaths directly attributable to infection. The Economist machine‐learning model based on official excess‐mortality data and more than 100 other statistical indicators, estimating 17.3 million deaths on November 21st, 2021, making it one of the most lethal pandemics in history.^2^ Prior to the current COVID‐19 pandemic, two other coronavirus pandemics had occurred since 2000: the severe acute respiratory syndrome (SARS) pandemic of 2002–2004, and the Middle East respiratory syndrome (MERS) pandemic that began in 2012 and of which cases still are sporadically reported.

The first COVID‐19 infection in Australia was detected on 25 January 2020 and occurred in a traveller who had arrived in Melbourne from Wuhan a week prior. The first death attributable to COVID‐19 occurred on 1 March 2020 and, as of the time of writing, Australia had reported over 72,000 cases and over 1,000 deaths.^3^ The initial exponential growth in case numbers prompted the Australian commonwealth, state, and territory governments to respond with sequential and increasingly strict control measures (Kompas et al., 2021). The emergence of a more infectious form of COVID‐19, the *Delta* (B.1.617.2) variant that had first been identified in India, and its arrival in Australia in May of 2021, led to a surge in cases necessitating further suppression measures (known as ‘lockdowns’) beginning in the state of New South Wales (NSW) but extending elsewhere. Rapid development of several vaccines directed against SARS‐CoV‐2, and their approval for public use by bodies such as Australia's Therapeutic Goods Administration (TGA) in mid‐February of 2021, marked the beginning of a transition from pandemic control by strict public health measures (test, trace, isolate, quarantine) to a broader approach including vaccination. Modelling of the effects of the pandemic on pre‐vaccination Australia compared different levels of public health measures with the extreme comparator of unmitigated and uncontrolled spread, concluding that the latter would have led to a total number of infections of 16 million and 260,000 deaths. An uncontrolled approach in a totally unvaccinated population would have seen Australian GDP fall by as much as 47.9 per cent (Kompas et al., 2021).

Moving forward, the plan for Australia – as it is with most other countries – is to overcome the health and economic effects of the COVID‐19 pandemic by population‐wide vaccination and finding a balance of public health measures. The ideal outcome of these measures is to allow the virus to become ‘endemic’ where the infection occurs in low and controllable numbers of people with intermittent controllable surges. If COVID‐19 became an endemic infection, however, it does not mean it would have no economic effect. Influenza surges occur on a yearly basis and sometimes more virulent strains cause local epidemics that have serious economic consequences (Mao et al., 2012).

Vaccines are among the most cost‐effective health technologies of all time: immunisation of a proportion of the population above a critical threshold level protects individuals and the community by preventing disease outbreaks (Wagner et al., 2020). Yet the premise that the population‐level health and economic effects of COVID‐19 infection can be controlled depends on a number of underlying assumptions: that vaccines are effective; that members of the community abide by public health measures such as ‘vaccine passports’; and, that a sufficient proportion of the population are vaccinated to allow suppression of new variants that could evade vaccine control. However, vaccine passports, which are one of the public health measures adopted by countries, have involved a plethora of local, provincial and national models that are not interoperable, leading to a chaos of different rules.^4^ Modelling of different public health measures suggests that, of all approaches, high vaccination levels is crucial to ending the COVID‐19 pandemic.^5^ However, as late as July of 2021, the WHO expressed concern that “vaccine inequity will have a lasting and profound impact on socio‐economic recovery.”^6^

Unfortunately, even in developed countries where the standard of healthcare is high and supply not an issue, it seems that a proportion of adults are refusing to be vaccinated against COVID‐19, or are expressing hesitation in accepting vaccination. The issue of vaccine hesitancy and refusal is so important that the OECD issued the following warning^7^:

“Many countries are observing increasing levels of distrust in government capacity to handle the crisis and implement coherent policies. This has resulted in declining compliance with public health‐related rules, and increasing scepticism about long‐term economic recovery. More broadly, the pandemic has triggered widespread disinformation that has undermined both understanding and acceptance of science and public policy, and this extends to the issue of vaccine acceptance. Despite widespread recognition that COVID‐19 is a critical issue to people all around the globe, many remain unwilling to be vaccinated.”

Vaccine hesitancy and refusal may undermine all other efforts to control the pandemic and, for this reason, could have major economic effects both on the Australian and global economies. In this paper we review the literature to ascertain the potential economic effects of vaccine hesitancy and, most importantly, vaccine refusal.

## II Pandemics and Economics

The outbreak of the highly contagious COVID‐19 pandemic overwhelmed health systems around the world and forced unprecedented containment measures: border closures and quarantines; stay‐at‐home orders; closure of retail stores and other businesses as well as schools and universities. This ‘pandemic shock’ had profound, ongoing economic consequences. A pandemic is defined by the World Health Organisation (WHO) as an “epidemic occurring worldwide, or over a very wide area, crossing international boundaries and usually affecting a large number of people.” It is important to understand, though, that the WHO definition does not take into account levels of population immunity, the nature of the infectious organism, or the severity of the disease (Kelly, 2011). The prototype pandemic was that of the global influenza epidemic of 1918/19 – the “Spanish ‘flu’” – that took the lives of 40 million people globally. One of the unique features of that pandemic was that it disproportionately affected prime aged women and men who had the highest mortality rate overall.^8^ The pandemic occurred in the aftermath of World War One, facilitated by international repatriation and demobilisation of soldiers, so isolating the economic impact of the pandemic itself has always proven challenging. Bishop, in an analysis^9^ for the Reserve Bank of Australia (RBA), concluded over the period 1918–21 that real GDP *per capita* fell by around 6 per cent globally and manufacturing output in the US reduced by 18 per cent.

Modern pandemics resulting from coronaviruses, such as the severe acute respiratory syndrome (SARS) of 2003 and the Middle East respiratory syndrome (MERS) of 2012 and 2015, spread rapidly due to travel and trade, urbanisation, and globalisation. Shang et al. (2021) reported these recent pandemics as having both short‐term fiscal impacts and longer‐term economic impacts. The direct costs of treating affected individuals include the provision of staffed health facilities and consumables such as personal protective equipment (PPE). Direct costs also include public health measures to curb infections: quarantines; standing‐up health facilities; isolation of infected cases; and contact tracing. More broadly, economic shocks result from shortages of labour due to illness, quarantine or supply such as through migration, disruption of transport networks, closure of workplaces, restrictions on trade and travel, as well as fear‐induced consumer behaviour. The SARS and MERS pandemics caused significant ‘pandemic shocks’ in affected countries due to demand side effects: particularly reductions in consumer spending in hotels and restaurants, and the transportation sector (Tanaka, 2021). The SARS pandemic principally affected China and Hong Kong, with the flow‐on demand shock also affecting other economically connected East Asian countries such as Taiwan and Singapore.^10^

## III Economic Effects of the COVID‐19 Pandemic

The effect of the COVID‐19 pandemic has been, and continues to be, one of the greatest economic shocks in Australia's history. One way to examine this is the ‘3Ps’ framework, which is commonly used to explain economic growth. The framework enables economic growth to be decomposed into the changes in the potential workforce, the share of that workforce that is employed, and how much they are generating from their work — population, participation and productivity, respectively. With borders effectively closed, the pandemic has reduced Australia's population growth rate^11^. The pandemic has also affected participation in the economy — with lockdowns much of the services sector (more than 80 per cent of economy) is effectively shut down. And so, the pandemic has also affected productivity — the amount of output per hour worked.

An analysis of the first phase of the pandemic by Lim et al. (2021) described widespread negative effects from international border closures affecting the tourism and higher education sectors and reducing migration; major effects on the labour market; and “a massive negative pervasive impact” on domestic demand and consumption. The most severe effects were felt by women, the young, and accommodation and food services, transport, arts and recreation, and retail trade.

In August of 2021 the Australian Commonwealth Department of the Treasury released an economic impact analysis^12^ of increasing vaccination rates based on modelling prepared for the National Cabinet by the Doherty Institute.^13^ That analysis concluded that it was more cost effective to minimise the number of COVID‐19 cases by taking early and strong action (that is, imposing lockdowns) in response to outbreaks of the Delta variant than allowing higher levels of community transmission which ultimately requires longer and more costly lockdowns. However, with rising vaccination rates fewer lockdowns and other restrictions would be required to minimise cases of COVID‐19 thus reducing the economic cost of managing the virus. Based on the Doherty modelling, at 80 per cent vaccination rates, averaged over the whole community, direct economic costs were likely to be approximately $140 million per week. The modelling assumed continuous application of low‐level restrictions rather than reliance on lockdowns. Despite this, Treasury warned that ongoing low‐level restrictions (primarily density and capacity constraints on workplaces and large events) would impose significant constraints, particularly on hospitality, arts and recreation and workplace environments. The reason was that, even at 80 per cent vaccination rates, it was likely that cases would need to be managed either by applying moderate lockdowns for 47 days per quarter, or by applying strict lockdowns for 29 days per quarter. A scenario of no lockdowns would require low levels restrictions for 81 days per quarter. At 80 per cent adult vaccination levels the Doherty Institute had estimated that it would be possible to manage outbreaks with low level restrictions in place 89 per cent of the time: alternatively, strict lockdowns could be imposed 31 per cent of the time. At a national level, Treasury estimates of the most economically cost‐effective managed transmission strategy, including baseline restrictions and the periodic low‐level lockdowns necessary to not overwhelm Australia's health system capacity, at a vaccination rate of 80 per cent, are of $590 million per week, and up to $1,260 per week per person. However, at vaccination rates of 70 per cent, the same strategy would cost $1.6 billion per week. Treasury did not model the economic costs of a severe and widespread outbreak that exceeds Australia's health system capacity. In that situation, the Treasury report stated, “it is expected that such a situation would carry very significant economic costs.”

## IV Vaccine Hesitancy and Refusal

**Figure 1:**
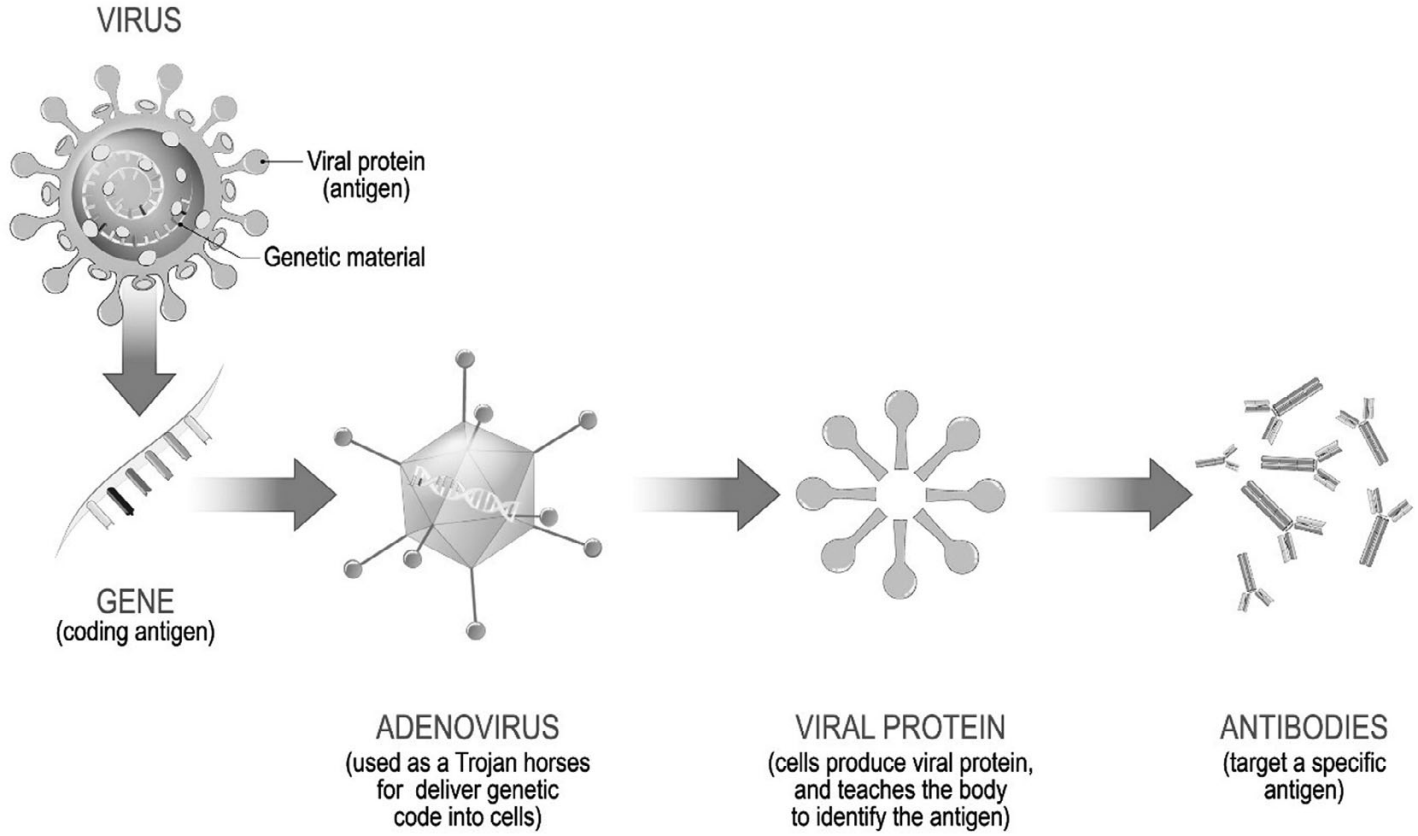
Mechanism of Action of the Oxford Vaccine (AstraZeneca).

**Figure 2:**
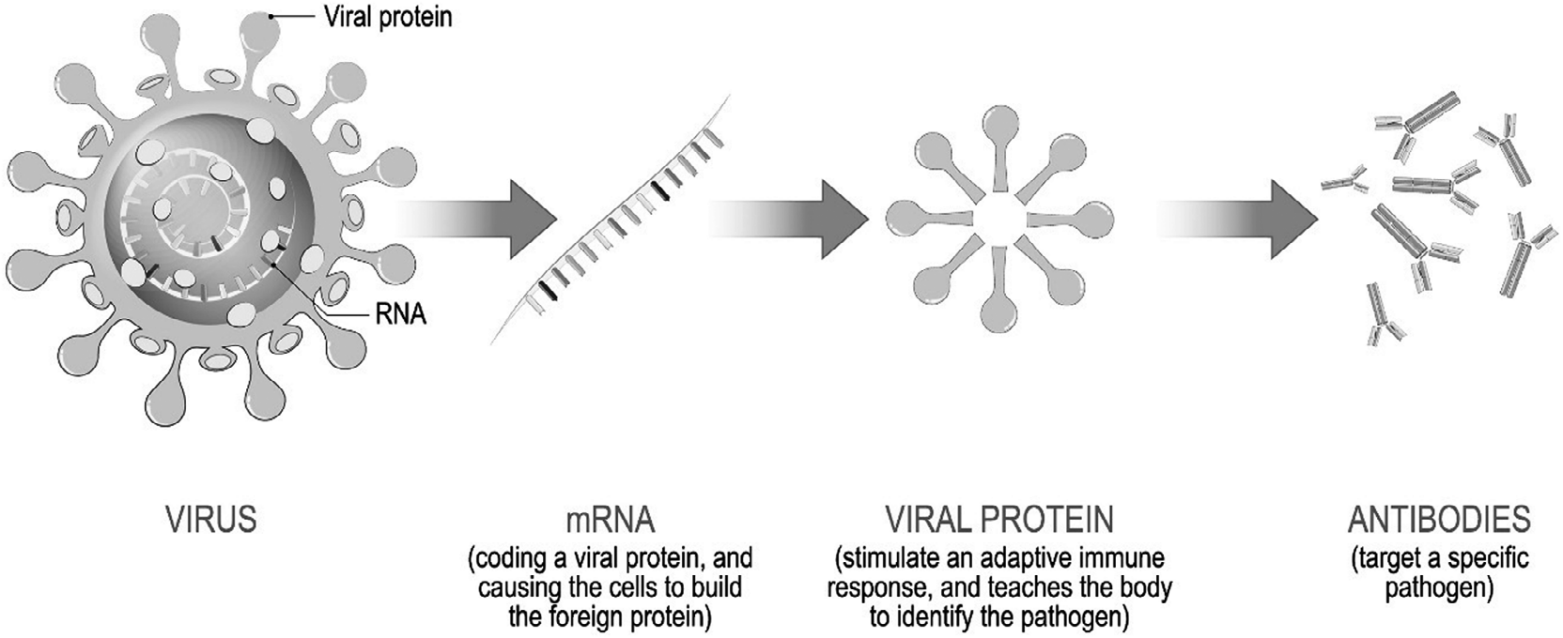
Mechanism of Action of the Newer mRNA‐Based Vaccines (Pfizer‐BioNTech, Moderna).

The Organisation for Economic Co‐operation and Development (OECD) states that the key to ending the COVID‐19 pandemic is an effective vaccination program that is co‐ordinated and equitable among and within countries, warning that failing to achieve this will threaten the global economic recovery.^14^ Remarkably, safe and effective vaccines against the virus were developed in very short timeframe – under one year (Gilbert & Green, 2021): development of a vaccine typically requires more than a decade, and prior to this pandemic the most rapidly developed vaccine was the mumps vaccine which took 4 years (Sassani et al., 1991). Because COVID‐19 results from a coronavirus similar that that of SARS and MERS, a substantial amount of research had already been performed in the last decade. At present there are two major types of vaccine available to Australians, the traditional type (developed by the Oxford group and manufactured under licence to AstraZeneca) (Fig. 1) and the newer technology of mRNA vaccines (manufactured by Pfizer and Moderna) (Fig. 2). Despite the rapidity with which these vaccines have been developed and brought to market the vaccines have met the usual rigorous guidelines for testing required by regulatory bodies such as the TGA and United States' FDA (Tau et al., 2021).

However, because COVID‐19 is a newly discovered virus and the vaccines that protect against infection have been available for such a short time, important questions remain about the duration of protection that occurs after either vaccination or infection, and about whether the emergence of new ‘variants’ of the coronavirus will evade vaccination. Variants are genetic changes in the virus that occur over time and alter its potential to cause disease and, importantly, the effectiveness of vaccination in preventing infection (Giovanetti et al., 2021). At the time of writing, the so‐called ‘Delta (B.1.617.2) variant’ had caused a global surge in infection, hospitalisation, and death (Twohig, et al., 2021) and the ‘Omicron (B.1.1.529) variant’ surge was peaking (Kannan et al., 2021). The emergence of new variants is a major driver of pandemics. Variants arise when large number of viral infections are present in the community, allowing more opportunity for subtle changes in the virus as it replicates. The importance of variants is that they may evade the immune response generated by vaccination, and if a variant is more contagious that existing forms of the virus, it can take over as the predominant pathogen. In a recent study from the US, fully‐vaccinated adults who became infected with the Delta variant required hospitalisation in 3.2 per cent of cases, intensive care (ICU) admission in 0.5 per cent of cases, and mechanical ventilation in 0.2 per cent of cases (Griffin et al., 2021).

**Figure 3:**
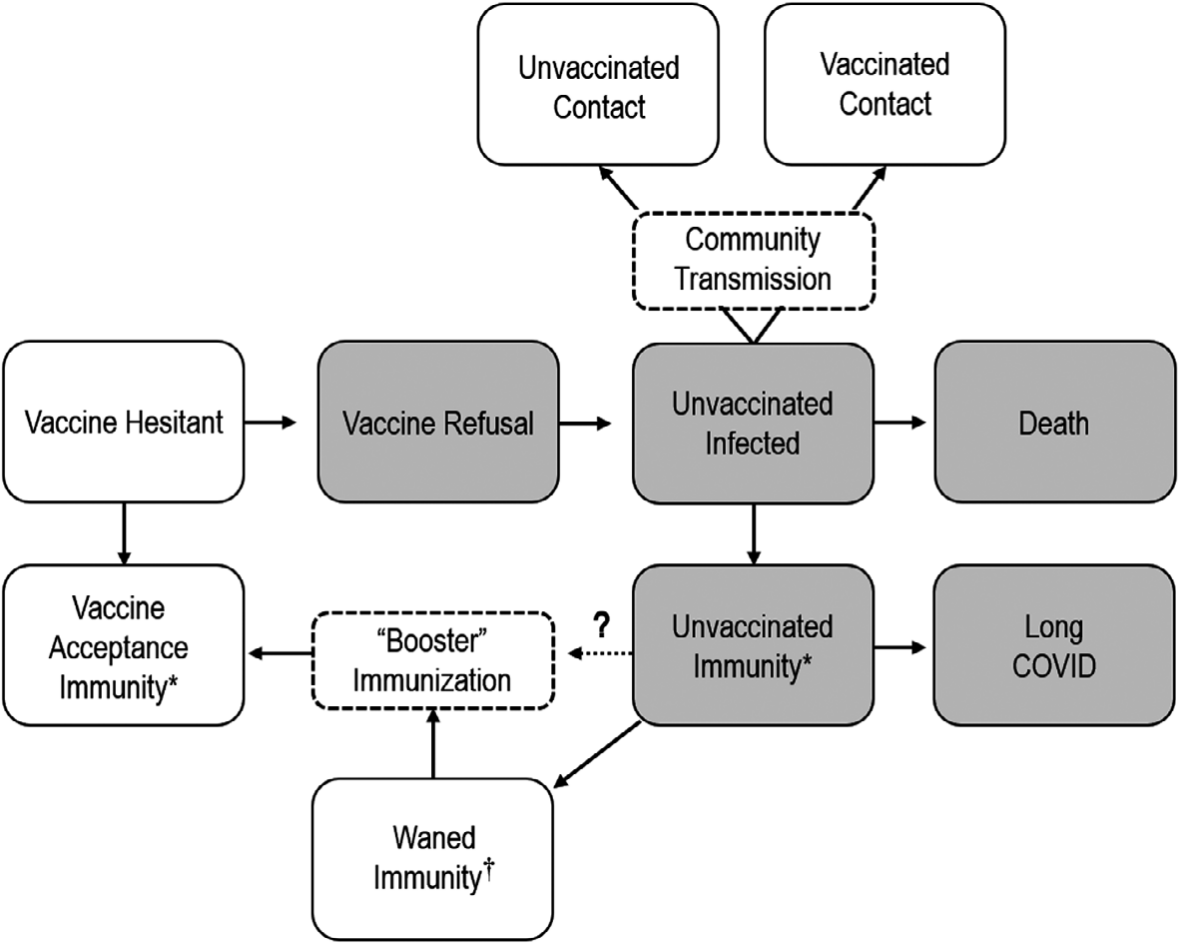
Schema for Understanding Potential Outcomes of Vaccine Hesitancy and Refusal. *The Level of Protection Provided by Immunisation and Infection Remain to Be Determined. ^†^The Duration of Immunity and Need for Booster Injections Remains Uncertain.

As developed countries' vaccination programs accelerate, the only people who remain unvaccinated are those 2–3 per cent with underlying conditions that make vaccination unsafe, (Shaker et al., 2021) and those who refuse vaccination. However, determining the level of vaccine cover necessary to allow resumption of normal economic activity is a complex task. Robust epidemiological and economic models are predicated on range of data and assumptions as shown in Figure 3. Some of the key uncertainties that affect projections can be summarised as follows:

1. What proportion of the vaccine hesitant population ultimately will accept vaccination?
2. Of ‘healthy’ vaccine refusers (as distinct from those who cannot be vaccinated due to underlying health problems) what proportion will contact the disease, and how many others will they infect?
3. Are there behavioural or other characteristics of vaccine refusers that make them more likely to transmit the virus?
4. Since vaccinated individuals can become infected, will they transmit the virus to others?
5. Will vaccine refusers who become infected change their behaviour, and will they have enduring immunity?
6. As the community rate of infection falls over time, will the perception of reduced risk provoke more vaccine refusal?
7. What proportion of the vaccinated will accept the need for ongoing booster vaccinations?

If COVID‐19 infections continue at current scale, new and more virulent variants will increase, with the potential to cause surges that threaten further economic disruption. Because of these uncertainties, a number of authorities now are questioning whether ‘herd immunity’ can be reached. In a review in *Nature*, Aschwanden surveyed expert opinion and concluded that it was unlikely that herd immunity thresholds would be reached.^15^ The consensus was that although community vaccination would have major benefits, new variants would arise and immunity would wane over time, leaving communities having to “battle the threat [and] deal with future surges.”

### (i) How Many People Are COVID‐19 Vaccine Hesitant?

Vaccine hesitancy and refusal are not new phenomena. The word ‘vaccine’ is derived from *Vaccinia*, the virus responsible for smallpox – a highly‐contagious viral infection estimated to have caused 400,000 deaths a year in 18^th^ century Europe and that had a fatality rate of close to 75 per cent (Riedel, 2005). While vaccination against smallpox was undoubtedly a public health triumph, contemporary anti‐vaccination campaigns were mounted based on arguments that sought to minimise the risk of the disease, exaggerate the risks of the vaccine, and play on public fears of government control.^16^ Sir William Osler, the so‐called ‘Father of modern medicine,’ was “so fed up with the ‘anti‐vaxxers’ of 1910 that he dared them to expose themselves to smallpox and promised to personally pay for the resulting funeral expenses.” (Swingle, 2018).

Survey data drawn from the Melbourne Institute's fortnightly *Taking the Pulse of the Nation* initiative^17^ estimated that, at the end of August 2021, approximately 12 per cent of adult NSW residents did not intend to have COVID‐19 vaccination. That proportion had actually increased from a level under 10 per cent as measured in July of 2021 – and despite a surge in cases in that state, associated public health orders, and a prolonged lockdown conditional on high community vaccination rates.^18^ As of the end of January 2020, the Australian Commonwealth Health Department reported that 93.3 per cent of Australians 16 years and older had two doses of a COVID‐19 vaccine.^19^ International studies report similar findings in high‐income countries. A study conducted in July 2021 of working age adults in France found that 29 per cent of respondents refused vaccination (Schwarzinger et al., 2021). A similar study conducted in the United Kingdom and the Republic of Ireland reported that 31 per cent of the study population were either hesitant or refused COVID‐19 vaccination (Murphy et al., 2021). These figures are high enough that public health experts now are expressing concern that vaccination levels may never be high enough to reach so‐called ‘herd immunity,’ where the proportion of susceptible individuals in a community is so low that community transmission does not occur. Ali Mokdad, Professor of Global Health at the University of Washington and head of that university's Institute of Health Metrics, said in a recent interview: “Vaccine hesitancy is a big problem for all of us… until now, the nationwide vaccine campaign has seen demand outstrip supply, but I believe that will soon change. We will have more vaccines than people willing to take the vaccine.”^20^

**Figure 4:**
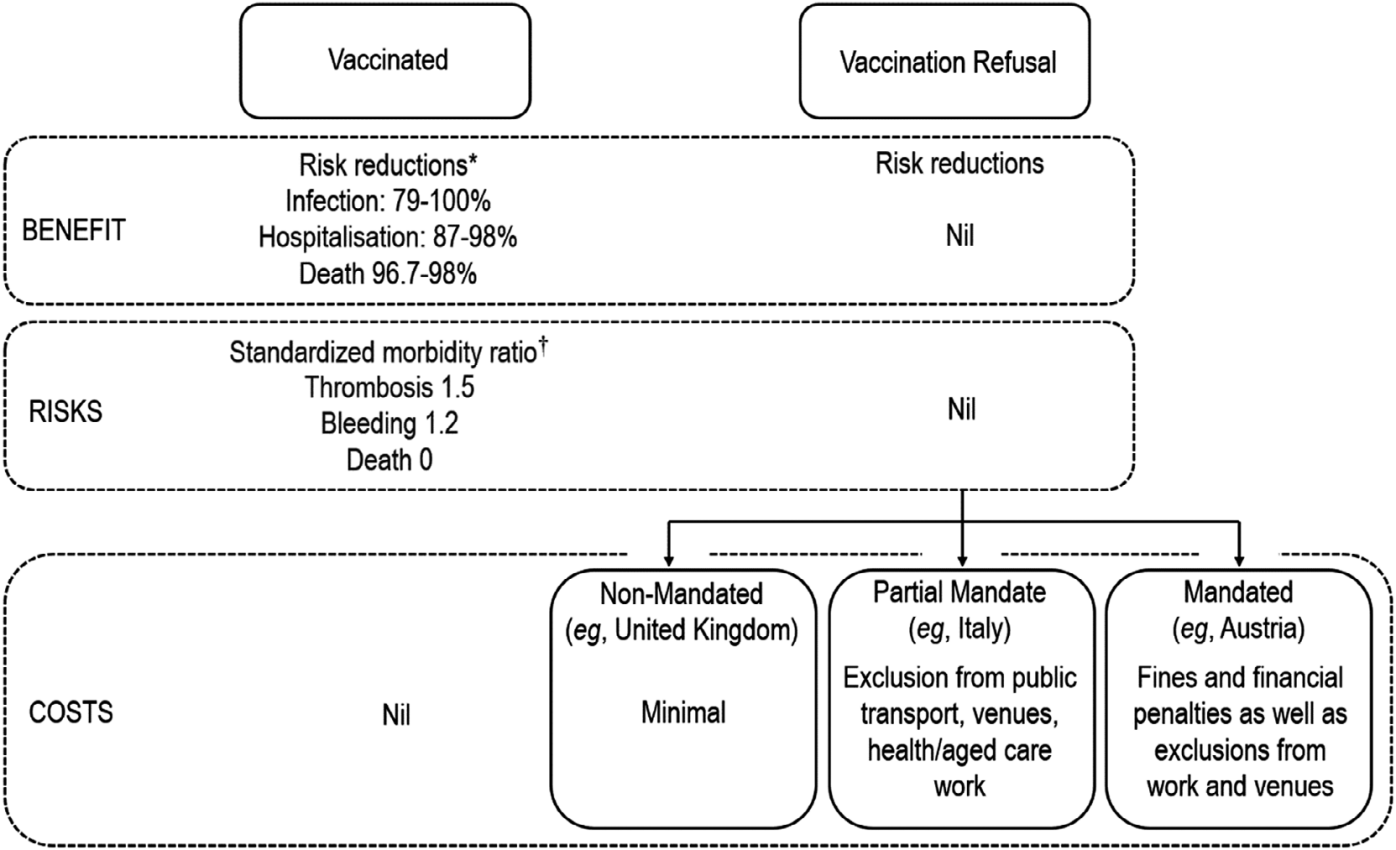
Schema for Understanding the Benefits, Risks, and Costs of Vaccination and Vaccination Refusal in the Prime Aged Population of a Country. The ‘Costs’ Layer Includes Data Accurate at the Time of Writing. *Estimates of Risk Reduction in Prime Aged Vaccine Recipients from Abd‐Elsayed and D'Souza (2022). Estimates of the Risk Ratios Attributable to Vaccination in Healthy Prime Aged Recipients from Pottegayrd et al. (2021).

In Australia, the subsequent emergence of the Delta variant of SARS‐CoV‐2 led to public health movement lockdowns from June 24^th^, 2021, in the most populous state of New South Wales, August 6^th^ in Victoria, the next most populous state, and August 12^th^ in the Australian Capital Territory, such that approximately 15 million Australians were in lockdown.^21^ It seems the Delta variant of SARS‐CoV‐2 outbreak in Australia galvanised the population to vaccinate, although further time will tell if actual vaccination exceeds the rates predicted by *a priori* hesitancy. However, as of the time of writing, Austria and The Netherlands, countries with high vaccine‐hesitancy and low vaccination rates, have instituted similar public health lockdowns due to Delta‐variant outbreaks, leading to considerable unrest, and whether vaccination rates will increase remains to be seen. A summary of the benefits, risk, and costs of vaccination and vaccine refusal is presented in Figure 4.

### (ii) Why Are People Vaccine Hesitant?

Sobkowicz and Sobkowicz (2021) present an excellent framework for understanding motivations and undercurrents that seem to be driving vaccine hesitancy and refusal:

“The impact of the pandemic on everyday life and the economy is significant on a global scale. The combination of fears related to health with the severe effects of social and economic lockdowns creates an emotional landscape in which distrust of the medical industry in general and anti‐vaccination arguments, in particular, can easily take hold. Moreover, the rush with which the COVID‐19 vaccines were developed, the demanding conditions for their transportation and storage, and problems with their accessibility and distribution open up a way for [anti‐vaccination] campaigns using rationally sounding arguments and appeals to safety and good practice. The situation is worsened by the fact that, in most countries, the reactions of governments and health authorities to the pandemic were, to say the least, less than optimal. In many countries, there were successive government blunders, as the authorities tried to balance health concerns with economic analyses. Wild swings between periods of lock‐down and loosening of restrictions signalled, to many of us, a lack of coherent strategies. This, in turn, created easy targets for anti‐establishment propaganda, facilitating attacks on the health authorities (including vaccine preparation and distribution processes).”

In their paper, *A behavioural economics perspective on the COVID‐19 vaccine amid public distrust*, Saleska and Choi (2021) have reviewed the recent literature on specific anti‐vaccination concerns about COVID‐19 vaccination. They note that “complex cognitive, social, and affective processes” guide decision‐making and that the speed with which the new vaccines were developed have prompted people “to express a desire to wait and see how it works for other people before getting it themselves.” This public heuristic is bolstered by pre‐existing cognitive biases: “the majority of Americans do not view the pharmaceutical industry in a particularly favourable light and, given the massive anticipated payoff for the pharmaceutical companies producing COVID‐19 vaccines, people may be even more inclined to believe that such companies do not have the public's interest in mind.”

Furthermore, recent vaccination policy‐related research in Germany indicates that mandatory vaccination laws may negatively impact on voluntary adherence, with increased social conflict and citizen distrust of government and professionals, which unfortunately leads to unavoidable enforcement (Schmelz and Bowles 2021). These German researchers advocate enhancing public trust in the vaccinations, and note this is likely to increase as more are vaccinated and infection rates fall. Finally, as appears to have been the case as above in Australia, reporting the prevalence of those vaccinated, may induce a cascade to abandon hesitancy.

### (iii) Socio‐Demographic Characteristics of the Vaccine Hesitant

Even small declines in vaccine coverage may have substantial public health and economic consequences (Lo & Hotez, 2017). For this reason the phenomenon of vaccine hesitancy and refusal has been a subject of study long before the COVID‐19 pandemic. In their comprehensive review published in 2020, Wagner et al., 2020 conclude that cognitive biases in risk perception are the main drivers: an overestimation of the risk of vaccine‐induced adverse effects, combined with misperceptions of lower individual vulnerability to the virus, and lesser overall severity of infection. Before the emergence of COVID‐19 low rates of adult immunisation against seasonal influenza and pneumococcal pneumonia were a cause of concern in Australia. In a 2020 policy white paper, the Australian Immunisation Coalition expressed concern that only 51 per cent of adults were adequately immunised against influenza and pneumococcal pneumonia, with rates particularly low for medically at‐risk populations.^22^

Because the potential negative effects of widespread COVID‐19 vaccine refusal now are so important, a number of studies have been conducted recently into the characteristics of the vaccine hesitant. An Australian online study of 3,000 participants ‐ of whom 7.2 per cent reported they ‘would probably not have the vaccine’ and 5.5 per cent said they definitely would not be vaccinated – found that refusal was associated with socio‐economic disadvantage, lower levels of education and being ‘religious’, and conservative voting intentions (Edwards et al., 2021). A study of 2,000 French adults undertaken in July of 2020 reported that vaccine refusal was associated with female gender, lower educational level, poor compliance with vaccination in the past, and not having an underlying health condition (Schwarzinger et al., 2021). A similar study of just over 3,000 adults in the UK and Republic of Ireland also reported the vaccine hesitancy to be associated with female gender, working age, urban residence, lower income level, and conservative voting intentions (Murphy et al., 2021). A more detailed analysis of UK respondents found that the vaccine hesitant had lower levels of trust in science, health care professionals, and scientists, as well as higher levels of conspiracy beliefs. Vaccine refusal appears to be more common in some ethnic minorities, such as Pakistani and Bangladeshi groups in the UK (Razai et al., 2021) and African‐American and Hispanic groups in the US (Khubchandani & Macias, 2021). Surprisingly, levels of vaccine hesitancy have been reported to be as high as 83 per cent in African‐American and Hispanic health care workers surveyed in the US (Momplaisir et al., 2021). There is a need to address mistrust among culturally and linguistically diverse communities that are likely at greater risk from COVID‐19, due to socio‐economic adversity and racism, such as Black communities in the US and diverse groups in Australia (Jaiswal et al., 2020; Looi et al., 2021). This mistrust has led to lower vaccination rates in marginalised and socioeconomically disadvantaged groups, such as the Aboriginal and Torres Strait Islander community in Australia, with only 69.3 per cent over 16 having had a first vaccination, and only 57.6 per cent being fully vaccinated, and is likely to be the case for similar groups in the US and UK.17a^23^.

### (iv) Social Media and Vaccine Hesitancy

Protests, ostensibly about vaccine mandates, have occurred regularly in Australia and overseas during 2021 and 2022, culminating in a major gathering in Canberra in February of 2022. These events were both promoted and ‘livestreamed’ on social media platforms. A strong role for social media disinformation campaigns has been identified as a driver of vaccine hesitancy and refusal and this trend has accelerated during the COVID‐19 pandemic. A study of the *Twitter*™ platform between September and December of 2020 reported that anti‐vaccination ‘tweets’ (using hashtags such as #vaccineskill and #vaccinesharm) were re‐tweeted 7.4 times more commonly than pro‐vaccine tweets and 31 times more than neutral tweets (Germani & Biller‐Andorno, 2021). Anti‐vaccination tweets received replies 13 times more commonly than other tweets. The authors concluded that, “anti‐vaccine users form a polarized network with little or no interaction with outsiders, in which users strengthen their positions by sharing each other's contents.” The role of social media in dissemination and amplification of anti‐vaccination content has been well‐recognised for some time. Puri et al. (2020) report that “social media dissemination of vaccine adverse events results in outbreaks of vaccine‐preventable illnesses with a more protracted course, lasting 150% longer.” Their review involving platforms such as *Facebook*™, *Instagram*™, and *Twitter*™ led them to comment that:

“It is not readily evident why social media is so disproportionately successful in promoting vaccine hesitancy as opposed to uptake. Social media users may represent a skewed population sample with baseline misperceptions regarding the benefits and side effects of vaccination whilst simultaneously lacking familiarity with the consequences of vaccine‐preventable disease.”

Unfortunately, agent‐based modelling of attempts to block or take down anti‐vaccine social media posts suggests that such ‘censorship’ is likely to provoke detrimental effects perceived as “coercive” with the risk of increasing polarisation and adoption of “persecution and martyrdom tropes.” (Sobkowicz & Sobkowicz, 2021).

The prominence of vaccine hesitancy and refusal in public discourse arises from what Kuran and Sunstein (1999) termed availability cascades – self‐reinforcing collective belief formation, through which a perception, in this case of risk from the vaccine, fuels a chain reaction *via* rising availability in public discourse of increasing plausibility (Kuran & Sunstein, 1999). In turn, cognitive negativity bias can enhance the awareness of risk, increasing the likelihood of social media propagation (Baumeister et al., 2001). Social media‐driven availability cascades of negative cognitions and perceptions can cause cognitive contagion, including fear (Kramer et al., 2014; Looi et al., 2021). Conspiracy beliefs that COVID‐19 and vaccination represents a secret plot by alliances of powerful individuals or organisations are prominent in social and other media, and are particularly difficult to counter, as experts are considered part of the conspiracy (Jaiswal et al., 2020; Looi et al., 2021). Sensitive communication, avoiding use of the term conspiracy beliefs, will be needed, including addressing inequity and racism (Looi et al., 2021). However, there may be a role for authorities to advise of the adverse mental health effects of excessive exposure to social media regarding the pandemic and associated social unrest, as there is strong evidence that more than 1 h of media consumption related to disasters and social unrest may lead to post‐traumatic symptoms, anxiety and depression in addition to the adverse effects of misinformation, disinformation, and encouragement of conspiracy theories (Looi et al., 2021). Distress, mental illness and unrest are likely to negatively impact upon the economic growth “3 Ps” of population, participation, and productivity.

## V Economic Considerations in COVID‐19 Vaccine Refusal

Before the availability of effective vaccines reliance was placed on social measures such as lockdowns, masking, hand hygiene, and social distancing to control the pandemic. As Moore et al. (2021) point out, though, while these measures proved “effective in reducing the healthcare burden compared to an uncontrolled outbreak, this is achieved to the detriment of the economy, education and many other societal factors.” An effective vaccination program is the only possible way of controlling the pandemic, minimising the health costs, and opening the economy. Gans (2021) describes vaccination programs as “a textbook example of a positive externality,” with government interventions required to reduce free riding. Yet achieving population‐level immunity against COVID‐19 is an enormous economic challenge, as it is complex to determine the level of community‐wide vaccination required to allow opening of the economy. Without complete opening of international borders Australia's economy remains stifled, yet slow vaccination programs in low‐ and middle‐income countries will compromise trade and travel, and will foster development of new variants that could overcome current vaccines (Excler et al., 2021). Modelling the economic effects of vaccination programs must take account of: (i) the costs of the vaccine, its transport and administration, including the cold chain infrastructure; (ii) the economic effects of infection (both the direct costs of providing medical care and the indirect costs of lost productivity in the short and long term); and (iii) the likely efficacy of the vaccine in preventing infection. To accomplish this, modelling must be dynamic (since the proportion of vaccinated individuals usually increases over time) and typically uses either an SIR (Susceptible, Infected, Removed) model or Markov chains (Cooper, 2007) that similarly employ a unidirectional SEIR (Susceptibility, Exposure, Infection, Recovery) model. SIR models allow the evolution of variables over time with the dynamic equations:

$$S\left(t+1\right)–S\left(t\right)=-\mathrm{\beta S}\left(t\right)I\left(t\right)$$

$$I\left(t+1\right)–I\left(t\right)=\left(\mathrm{\beta S}\left(t\right)–\gamma \right)I\left(t\right)$$

$$R\left(t+1\right)–R\left(t\right)=\mathrm{\gamma I}\left(t\right)$$

**Figure 5:**
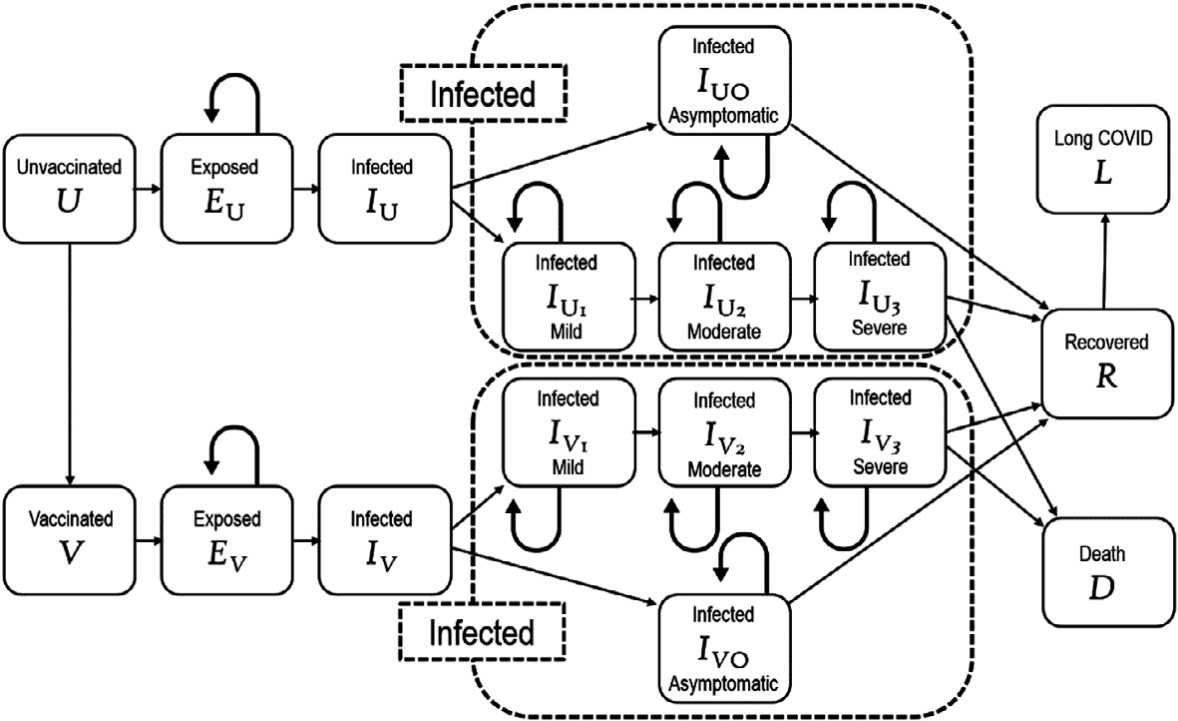
Example of a Typical Markov Chain Model Used to Evaluate the Economic Effects of COVID‐19 Vaccination Programs. This is Typical of the Models Used by Padula et al. (2021) and Wang et al. (2021a, 2021b).

Where *S*(*t*), *I*(*t*), and *R*(*t*) are the relative proportions of the population susceptible, infected, or removed (recovered or dead) respectively, and *γ* is the probability that an infected person will be removed in any given period while *β* is the probability that a susceptible person will be infected in a given period. An example of the complexity of Markov models is shown in Figure 5. To avoid key biases such models typically have to be age‐structured, with an example of this pertaining to modelling of the Delta variant outbreak in Australia by Chu et al. (2021). The outcomes typically are calculated using a human capital approach as described by Robinson (1993) and in quality‐adjusted life years (QALYs) (Hall & Viney, 2021).

The difficulty in economic modelling related to population‐level vaccination programs lies in current uncertainties about the true efficacy of the vaccine. How likely is it to protect from becoming infected with new variants? Even if a vaccinated individual becomes infected and does not become unwell, can they infect others? How long does a vaccination last? What proportion of the population will be either ineligible to receive a vaccination or refuse to be vaccinated? Are there fundamental behavioural or sociological characteristics of people who refuse vaccination that might make them more likely to infect others? If individuals who refuse vaccination become infected and recover, will they become immune to a satisfactory degree and will their immunity endure? Will a falling infection rate in the community alter population behaviour and make people less likely to accept vaccination? These are all important questions with economic consequences that must be addressed.

Economic models published before emergence of the delta and subsequent variants of COVID‐19 reported that one dollar invested in a vaccine would return between $13 and $28 in healthcare and education costs, and between $176 and $443 in statistical life (Wang et al., 2021a, 2021b). Padula et al.’ (2021) projected that productivity losses due to non‐vaccinated individuals in the labour market would total $15 billion in the US alone despite an overall reduction in COVID‐associated costs of 80 per cent due to vaccination alone.

The emergence of new variants of COVID‐19 is a key issue affecting economic modelling. International data show that existing vaccines were 94 per cent effective in preventing symptomatic infection of the original virus, but this efficacy drops to less than 64 per cent after the Delta variant becomes predominant, in fully‐vaccinated individuals (Baraniuk, 2021). Even vaccinated adults have waning levels of immunity within a year of immunisation (Anderson et al., 2020). Individuals who refuse vaccination are at risk of severe disease if they become infected: a study from California reporting that of unvaccinated adults who are infected, 7.6 per cent require hospitalisation, 1.5 per cent require admission to an intensive care unit, and 0.5 per cent require mechanical ventilation (Griffin et al., 2021). While infection confers immunity, the duration and strength of immunity remains unclear and susceptibility to new variants is unknown, as it is for the vaccinated (Wang et al., 2021a, 2021b). In addition, it now appears that as many as 70 per cent of adults who recover from acute COVID‐19 will suffer a constellation of ongoing health problems known as ‘“long COVID,” characterized by fatigue, muscle ache, breathlessness, and headache, with dysfunction of the heart, lungs, kidneys and liver (Iacobuccu, 2020). At the time of writing 40 adult “long COVID clinics”’ were operating in the UK, and this long term effect will affect productivity (an indirect economic effect) and requiring ongoing health care (a direct cost).

If a sufficient proportion of adults prove resistant to public health campaigns and legislative incentives and refuse to be vaccinated, this has the potential to undermine the effectiveness of population vaccination and threaten economic recovery at a global level. The socio‐demographic profile of the vaccine hesitant and refusers means they are likely to be over‐represented in occupations such as transport and service, threatening logistic chains and manufacturing. Vaccine hesitancy and refusal have the potential to promote ongoing surges in infection, placing strains on healthcare resources meaning that people with non‐COVID illnesses and conditions face delays in treatment and thus lower productivity. Infection surges themselves promote development of new variants.

### (i) Vaccine Refusal and Employment

Occupational transmission of COVID‐19 is important from two perspectives. In the first instance, workplaces present a risk for transmission with infection‐related mortality increased among men in the lower‐skilled occupations, and security guards have the highest death rate. Other occupations with increased risks included taxi and other drivers, restaurant chefs, sales and retail assistants, and those providing social care (Burdorf et al., 2021). Workplace transmission of infection has negative economic consequences dependent on the nature of the business: temporary closure of businesses affects supply chains, for example, or infected transport workers may transmit COVID‐19 in other geographical areas or across borders. Additionally, employees who contract the infection in the workplace might have legal grounds to sue their employer for damages.^24^ However, the pragmatic response to workplace transmission ‐ mandating vaccination against COVID‐19 ‐ has an uncertain legal status both in Australia^25^ and elsewhere in high‐income countries. The typical characteristics of vaccine refusers (low skilled occupations and lower socio‐economic status) mean they are very likely to be employed in these at‐risk occupations creating potential labour market economic consequences either through involuntary unemployment, compliance costs for business, or a combination of both.

## VI Conclusion

The global economic shock resulting from the COVID‐19 pandemic has imposed an unprecedented public health and socio‐economic burden on this generation. The pandemic and its effects can only be overcome through an effective global immunisation program. Safe and effective vaccines have been developed in record time and global vaccination programs are being rolled out. However, the virus itself is prone to structural changes that lead to new variants, some of which – such as the Delta variant – are more resistant to vaccination and cause higher rates of illness and death. As long as the virus spreads and mutates in the population, the risk of infection surges or emergence of new variants remains. A situation of ‘herd immunity’ ‐ where proponents predict that with a large proportion of the community ‘immune’ to COVID‐19 infection will mean there is little risk of ongoing surges – is unlikely, as shown in modelling by Chu et al. (2021). In developed countries, it is likely that the most common reason for suboptimal vaccination rates will be vaccine hesitancy and refusal. This is not a new phenomenon, but has become more entrenched and widespread with social media misinformation and disinformation campaigns, especially in marginalised and economically disadvantaged groups. This has enormous potential economic consequences at a global level. The “3 Ps” of population, participation, and productivity are foundering under the strain of the pandemic. If the COVID‐19 pandemic is to be overcome it is critical that vaccine hesitancy and refusal is understood and dealt with as a matter of urgency. Central to economics, but warranting further research and discussion, is the separate consideration of whether policy levers, such as financial incentives such as subsidies, rebates, tax relief, or penalties may be one of a range of suitable approaches to address hesitancy and refusal.

## Conflicts of Interest

The authors have declared no conflicts of interest.

## Footnotes

^1^
The WHO tracking website provides regular global data and can be accessed at https://covid19.who.int/

^2^
The Economist‐ COVID‐19 data – the pandemic's true death toll which can be accessed at: https://www.economist.com/graphic‐detail/coronavirus‐excess‐deaths‐estimates

^3^
The Australian Commonwealth Health Department publishes contemporaneous data on COVID‐19 infections accessible at: https://www.health.gov.au/news/health‐alerts/novel‐coronavirus‐2019‐ncov‐health‐alert/coronavirus‐covid‐19‐case‐numbers‐and‐statistics

^4^
Why vaccine passports are causing chaos, accessible at: https://www.economist.com/international/why‐vaccine‐passports‐are‐causing‐chaos/21805939

^5^
Modelling conducted for the University of Pennsylvania by Arnon and Rico, accessible at: https://budgetmodel.wharton.upenn.edu/issues/2021/3/22/health‐and‐economic‐effects‐of‐reducing‐covid‐19‐vaccine‐hesitancy

^6^
World Health Organisation: Vaccine inequity undermining global economic recovery, published July 22, 2021. Accessible at: https://www.who.int/news/item/22‐07‐2021‐vaccine‐inequity‐undermining‐global‐economic‐recovery

^7^
OECD, May 2021: Enhancing public trust in COVID‐19 vaccination. Accessible at: https://www.oecd.org/coronavirus/policy‐responses/enhancing‐public‐trust‐in‐covid‐19‐vaccination‐the‐role‐of‐governments‐eae0ec5a/

^8^
Brainerd, E. and Siegler, M.V. The economic effects of the 1918 influenza pandemic. Discussion paper number 3791. For the *Centre for Economic Policy Research*, London. Published February 2003.

^9^
Bishop, J. (2020), ‘Economic effects of the Spanish flu.’ Accessible at: https://www.rba.gov.au/publications/bulletin/2020/jun/economic‐effects‐of‐the‐spanish‐flu.html

^10^
Australian Treasury. (2003), ‘The economic impact of Severe Acute Respiratory Syndrome (SARS).’ Accessible at:

^11^
Treasury. (2020), Budget Paper No.1; Statement 2: Economic Outlook, p.2–7.

^12^
Australian Treasury: National Plan to Transition to Australia's National COVID‐19 Response Economic Impact Analysis, 3 August 2021 Accessible at: https://treasury.gov.au/sites/default/files/2021‐08/PDF_Economic_Impacts_COVID‐19_Response_196731.pdf

^13^
The Doherty Institute Modelling Report to advise on the National Plan to transition Australia's National COVID Response, 3^rd^ August 2021. Accessible at: https://www.doherty.edu.au/uploads/content_doc/DohertyModelling_NationalPlan_and_Addendum_20210810.pdf

^14^
The OECD statements on COVID‐19 are accessible at: https://www.oecd.org/coronavirus/en/vaccines

^15^
Aschwanden, C. (2021), ‘Five reasons when COVID herd immunity is probably impossible.’ *Nature*, published online 18^th^ March. Accessible at: https://www.nature.com/articles/d41586‐021‐00728‐2

^16^
Described in detail by Larsson in “COVID‐19 anti‐vaxxers use the same arguments from 135 years ago” accessible at: https://theconversation.com/covid‐19‐anti‐vaxxers‐use‐the‐same‐arguments‐from‐135‐years‐ago‐145592

^17^

https://melbourneinstitute.unimelb.edu.au/data/covid‐19‐tracker

^18^

https://melbourneinstitute.unimelb.edu.au/publications/research‐insights/ttpn/vaccination‐report

^19^

https://www.health.gov.au/sites/default/files/documents/2022/01/covid‐19‐vaccine‐rollout‐update‐jurisdictional‐breakdown‐31‐january‐2022.pdf

^20^
Quoted in Brumfiel (2021): Vaccine refusal may put herd immunity at risk, researchers warn. Accessible at: https://www.npr.org/sections/health‐shots/2021/04/07/984697573/vaccine‐refusal‐may‐put‐herd‐immunity‐at‐risk‐researchers‐warn

^21^
Australian Government COVID Shield – COVID‐19 Vaccine Rollout. Accessible at: https://www.health.gov.au/sites/default/files/documents/2021/11/covid‐19‐vaccine‐rollout‐update‐21‐november‐2021.pdf

^22^
Enhancing adult vaccination coverage rates in Australia. Policy white paper, 2020. Accessible at: https://www.immunisationcoalition.org.au/enhancing‐adult‐vaccination‐coverage‐rates‐in‐australia/

^23^
Australian Government COVID Shield – COVID‐19 Vaccine Rollout. Accessible at: https://www.health.gov.au/sites/default/files/documents/2021/11/covid‐19‐vaccine‐rollout‐update‐21‐november‐2021.pdf

^24^
A University of NSW analysis of workplace obligations and law, “Can you sue your employer if you contract COVID‐19 in the workplace?” is accessible at: https://www.unsw.edu.au/news/2020/09/can‐you‐sue‐your‐employer‐if‐you‐contract‐covid‐19‐in‐the‐workpl

^25^
A contemporary analysis of the legality of mandatory vaccination in Australia by Professor Joo‐Cheong Tham of the University of Melbourne Law School is accessible at: https://theconversation.com/can‐australian‐employers‐make‐you‐get‐a‐covid‐19‐vaccine‐mostly‐not‐but‐heres‐when‐they‐can‐165755
20a Johns Hopkins Coronavirus Information Centre – Understanding vaccination progress, Accessible at: https://coronavirus.jhu.edu/vaccines/international
